# Prosthetic valve thrombosis – association of genetic polymorphisms of *VKORC1*, *CYP2C9* and *CYP4F2* genes

**DOI:** 10.1097/MD.0000000000014365

**Published:** 2019-02-08

**Authors:** Kalpana SR, Bharath G, Simran Jain, Nagaraja Moorthy, Satvic C. Manjunath, Rita Christopher

**Affiliations:** a Sri Jayadeva Institute of Cardiovascular Sciences & Research, Depatmment of Pathology; b Sri Jayadeva Institute of Cardiovascular Sciences & Research, Department of Cardiology, Bannerghatta Road, 9th block Jayanagar, Bangalore - 69, India; c National Institute of Mental Health and Neuroisciences, Department of Neurochemitry.

**Keywords:** genetic polymorphisms, mechanical heart valves, prosthetic valve thrombosis

## Abstract

Prosthetic Valve Thrombosis (PVT), in spite of the advances in the valve design and the material used, remains a serious complication of mechanical cardiac valve replacement. The factors influencing the development of PVT are: thrombogenicity of the valve, hemodynamics of the transprosthetic blood flow and ineffective anticoagulation. Genetic polymorphism of the genes *VKORC1* (-1639 G > A and 1173 C > T), *CYP2C9* (∗2 & ∗3 alleles) and *CYP4F2* (1347 G > A) are known to influence the anticoagulant dose-effect response. Since there has not been any earlier study on the direct influence of gene polymorphism on the development of PVT, we investigated into this association.

Genotyping for the genes *VKORC1*, *CYP2C9* and *CYP4F2* was carried out by conventional PCR-RFLP method for 91 consecutive PVT patients. Subjects of our earlier study served as controls (n = 136).

Female patients and patients with smaller prosthetic valve size were more prone to developing PVT (68%, n = 62). Patients bearing A allele of *CYP4F2* 1347 G > A polymorphism exhibited a fivefold increased risk of PVT (OR = 5.022 (1.39–18.04), *P* = .013). G allele of *VKORC1* when analyzed in combination of genotypes showed a fourteen fold increased risk for developing PVT (OR = 14.25 (5.52–36.77), *P* = 0.001). *CYP2C9* (∗2&∗3) gene polymorphism did not show any significant association with PVT (OR = 1.54 (0.128 – 18.82), *P* = .731).

Patients bearing A allele of *CYP4F2* showed an increased risk of developing PVT in our case – control study.

## 1 Introduction

Prosthetic Valve Thrombosis (PVT) is a rare but serious complication of mechanical heart valve and is associated with significant mortality and morbidity.^[1]^ PVT is an obstruction of a prosthetic valve by non infective thrombotic material. The occurrence of PVT depends on the thrombogenicity of the prosthetic valve, hemodynamic of the transprosthetic blood flow and ineffective anticoagulation. Coexisting pro-thrombotic factors in the patient also predispose to PVT.^[2]^ Inherited disorders have also been reported to cause PVT like fibrinogen 455G > A polymorphism and MTHFR 1298 A > C.^[3]^ Despite improvements in the design of the prosthetic valves, the incidence of PVT in mechanical valves has been reported as 0.5% to 8%, in mitral and aortic positions.^[4]^ The risk of thromboembolism in these patients is said to be 1% to 2% even with the oral anticoagulation therapy.^[4]^

Being a tertiary cardiac care center with almost 500 valve replacement surgery in a year for rheumatic valvular disease, PVT is one of the common emergencies treated in our hospital, despite effective anticoagulation therapy. We have observed in a good number of cases, that there is no correlation of PVT with the International Normalized Ratio (INR) at the time of admission, nor is it associated with any other hypercoagulable states.

The polymorphisms of *VKORC1*, *CYP2C9* and *CYP4F2* genes are known to influence the mean dose requirement of vitamin K antagonists; acenocoumarol and warfarin^[5,6]^ of which *VKORC1* and *CYP2C9* are major determinants. The *VKORC1* gene is located on chromosome 16p11.2 and encodes the vitamin K epoxide reductase gene which catalyses the rate limiting step in vitamin K recycling. The anticoagulant drug functions by inhibiting this enzyme. The single nucleotide polymorphism (SNP) -1639G > A at promoter region or 1173C > T at intron 1 influences the anticoagulant dose requirement. The heterozygous and variant genotypes require a low dose of the anticoagulant when compared to the wild type, owing to the reduced activity of vitamin K epoxide reductase enzyme.^[7]^

*CYP2C9* is one of the most important Cytochrome P450 enzymes in the liver responsible for metabolizing clinically important drugs. The coumarin group of drugs is metabolized by *CYP2C9* enzyme*. CYP2C9* gene is located on chromosome 10q24.2. The allelic variants, *CYP2C9∗2* and *CYP2C9∗3* are known to result in the reduced functioning of the enzyme and hence, have an increase risk of bleeding complications.^[7]^

*CYP4F2* is a vitamin K1 oxidase in the liver and catalyses vitamin K1 to hydroxylated vitamin K1. The gene is located on chromosome 19p13.11. An SNP of *CYP4F2* gene (rs2108622, p.V433 M) results in reduced activity of the enzyme, influencing the anticoagulant dose requirement. It has an antagonistic effect as compared to *VKORC1,* wherein the carriers of variant allele require a higher dose of the anticoagulant compared to the wild allele.^[8]^

In our literature search, we have not found any study investigating into the direct association of *VKORC1*, *CYP2C9* and *CYP4F2* gene polymorphisms with the incidence of PVT. Hence, this is the first study to elucidate the direct influence of these gene polymorphisms, if any, on the occurrence of PVT.

## 2 Materials and methods

This was a prospective case control study conducted at Sri Jayadeva Institute of Cardiovascular Sciences and Research (SJICR), Bengaluru, India in collaboration with National Institute of Mental Health and Neurosciences (NIMHANS), Bengaluru, India. The Ethics committee of SJICR approved the study (Ethics committee registration number – ECR/423/Ins/KA/2013). This study conformed to the principles outlined in the Declaration of Helsinki.

### 2.1 Study subjects

Ninety-one consecutive South Indian patients admitted with the diagnosis of prosthethic valve thrombosis to PVT emergency cardiac care unit at SJICR, Bengaluru were recruited for the study, after carefully considering the inclusion and exclusion criteria. All the cases of PVT are diagnosed by transthoracic echocardiography followed by transoesophageal echocardiography.

### 2.2 Inclusion criteria

- Consecutive patients with diagnosis of prosthetic mechanical valve thrombosis on oral anticoagulation with acenocoumarol or warfarin.

The following patients were excluded from the study

- Patients aged <18 years
- Drug defaulters
- Patients with bioprosthetic valve implantation
- Patients on following medications –antiepileptics, including phenytoin and carbamazepine, antituberculous treatment
- Patients reactive to Human Immunodeficiency Virus (I & II), Hepatitis B or C and Syphilis (Venereal Disease Research Laboratory test) were also excluded.

### 2.3 Controls

Age and gender matched subjects from our earlier ICMR project (Project No. 5/4/ 1-7/12-NCD-II) served as controls for this study.136 chronic Rheumatic Heart Disease (RHD) patients of south Indian origin, who underwent valve replacement surgery with mechanical valve, were recruited for the study. Only those Patients receiving acenocoumarol therapy and maintaining a stable therapeutic INR between 2 to 3.5 for at least 3 months at SJICR were included as controls. Of the 136 cases, 27 patients had aortic valve replacement, 79 mitral valve and 30 had double valve replacement. A detailed history and also clinical finding from case files ruled out PVT or any other incidence of thrombosis in the controls.

### 2.4 Data and sample collection

Clinical details and blood samples were collected after obtaining written informed consent from all patients, both cases and controls. A detailed history of diet, comorbidity, medication, episodes of bleeding, or thromboembolism, if any, was collected.

Venous blood was collected in two Ethylenediaminetetraacetic acid (EDTA) vacuum tubes (5 ml) for complete haemogram and genotyping. One tube of 3.8% tri sodium citrate was collected for Prothrombin Time /International Normalized Ratio (PT/INR) estimation. Samples were collected from all the study cases before starting the thrombolytic treatment.

Complete haemogram was performed using an automated hematology analyzer (Coulter Ac.T-5 part differential cell counter, Beckman Coulter, CA).

PT/INR analysis was done using fully automated coagulation analyzer (Stago-compact STA, Neoplastine ISI 0.93, Stago, France) using platelet-free plasma obtained by centrifugation at 2000 g for 10 minutes at 25°C. Plasma was separated from the EDTA sample and stored at −80°C until further analysis.

**Figure 1:**
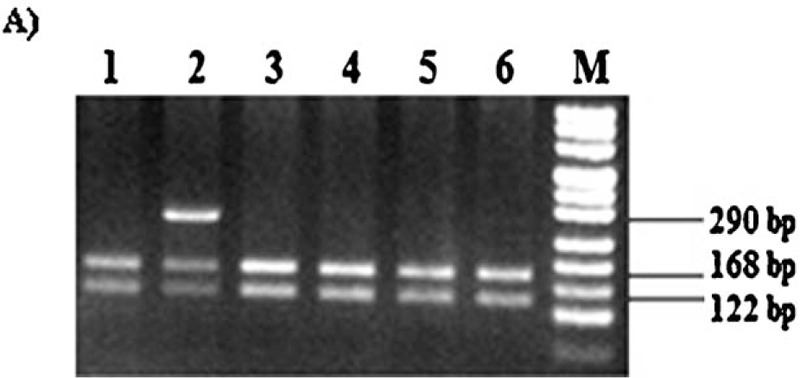
(A) RFLP analysis representing *VKORC1* -1639 G > A polymorphism samples digested with *MspI*. Lanes 1, 3, 4, 5, and 6 - wild (GG) genotype; lane 2 - heterozygous (GA) genotype; lane M – DNA marker 50 bp gene ruler (ThermoFisher Scientific).

**Figure 2:**
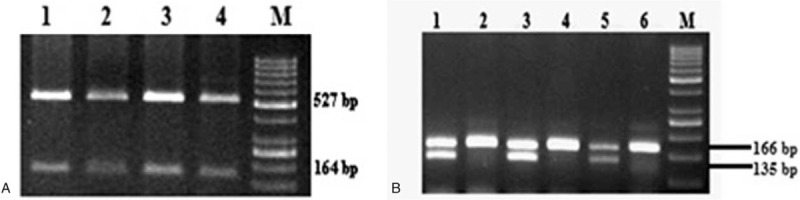
Gel pictures (A) & (B) depicting *CYP2C9* ∗2 & ∗3 polymorphisms respectively. (A) RFLP analysis of representative individuals with homozygous genotype digested with *AvaII* at lane 1, 2, 3, 4, and lane M – DNA marker GeneRuler 50 bp (ThermoFisherScientific). (B) RFLP analysis of representative individuals with homozygous genotype digested with *KpnI* (lane 2, 4, and 6) or heterozygous genotypes (lane 1, 3, and 5) *NsiI* and lane M- DNA marker GeneRuler 50 bp (ThermoFisherScientific).

Genomic DNA was extracted from peripheral blood using conventional phenol-chloroform method^[9]^ and quantified using NanoDrop 2000 (Thermo Fisher Scientific, Wilmington, Delaware). Genotyping for *VKORC1* (-1639 G > A), *CYP2C9* (∗2 &∗3) and *CYP4F2* (1347 G > A) polymorphisms was performed for all samples (91 cases and 136 controls). Genotyping of *VKORC1* (-1639 G > A) (Fig. 1) and *CYP2C9* (∗2 &∗3) (Fig. 2) were done using standard protocols as described in our previous study.^[10]^

### 2.5 Genotyping of *CYP4F2* (1347 G > A) polymorphism

**Figure 3:**
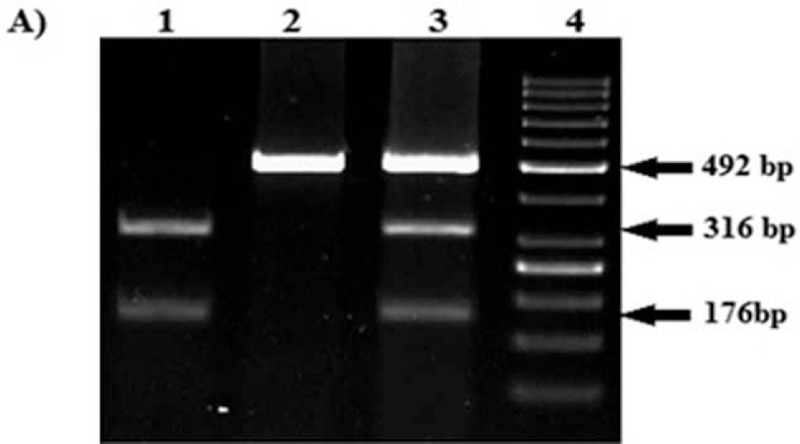
(A) Restriction fragment Length Polymorphism (RFLP) and representative band patterns of *CYP4F2* 1347G > A polymorphism samples digested with *Pvu-II-HF* (New England Biolabs), Lane1: Homozygous wild type (GG), Lane2: Homozygous mutant (AA), Lane 3: Heterozygous genotype (GA) and lane 4: Gene ruler 50 bp ready to use DNA ladder (ThermoFisher Scientific).

Genotypes of *CYP4F2* 1347 G > A (rs2108622) were detected by the protocol described by Deng et al^[11]^ A 492-base pair (bp) DNA fragment containing G > A substitution at 1347 nucleotide position was amplified using polymerase chain reaction (PCR) and cleaved by restriction enzyme *PvuII* (New England Biolabs, Massachusetts). *PvuII*- digested products were separated on 2% agarose gel. The band pattern obtained for wild homozygous (GG) was 316 and 173 bp. Heterozygous (GA) showed an intact band of 492 bp along with 2 cleaved bands of 316 and 173 bp, whereas homozygous mutant (AA) showed undigested intact band of 492 bp (Fig. 3).

### 2.6 Anti-phospholipid antibodies

The 2–3 ml of whole blood was collected and serum was separated by centrifugation. The serum samples were stored at −80°C until further analysis. Anti-Phospholipid antibody screening (Autobind; Tosho India Pvt. Ltd.) was performed for the quantitative determination of antibodies- class Ig M and Ig G.

### 2.7 Statistical analysis

Statistical analysis was performed using SPSS statistical package, version 21.0 (Corporation, NY). Categorical data are expressed in percentages. Continuous variables are expressed, as the mean ± SD. *P* value ≤.05 was considered statistically significant. Genotype frequencies were determined using standard frequency analysis and deviations of allelic frequencies from Hardy-Weinberg equilibrium was evaluated by allele counting method and χ^2^ test. Odds ratio (OR) was used to evaluate the odds of different genotypes. Binary logistic regression analysis was done to assess the relative risk for developing PVT in carriers of wild and variant alleles.

## 3 Results

### 3.1 Demographic and clinical characteristics

A total of 91 RHD patients with prosthetic valve thrombosis (29 males and 62 females) were studied. Demographic and clinical characteristics of patients are given in Table 1. Their mean age was 37.6 ± 11.0 years (range = 18–68 years). Smoking and alcohol consumption was noted in 4.39% of the cases.

**Table 1:**
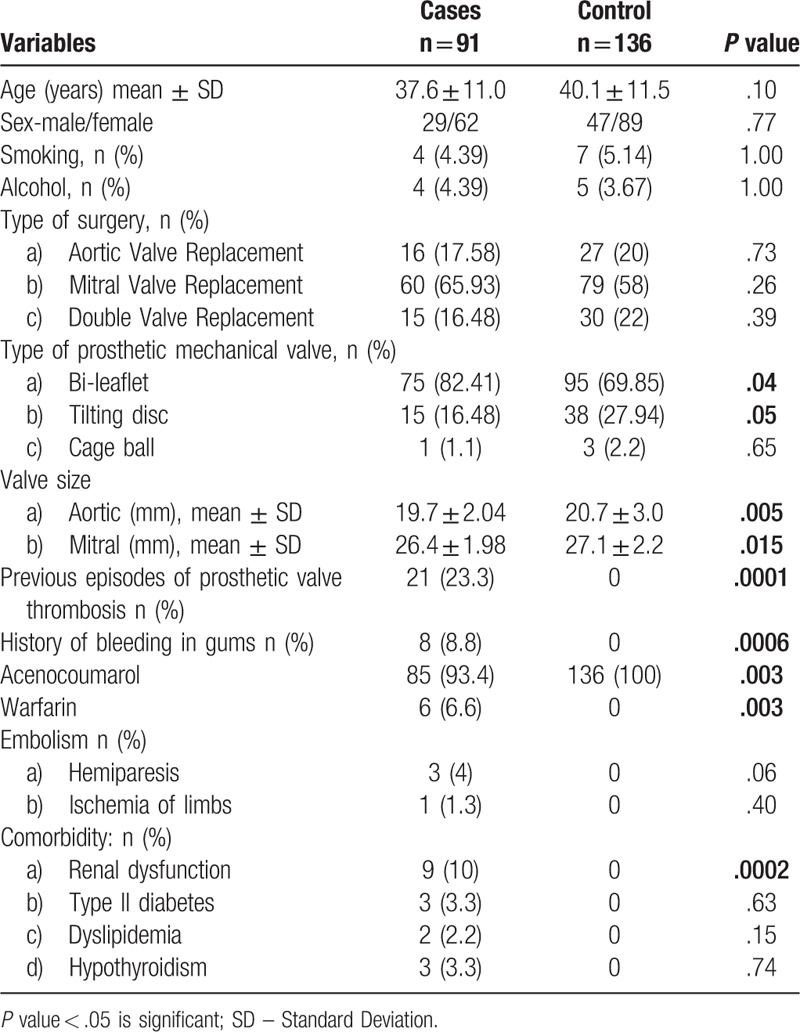
Demographic table of patients with mechanical cardiac valves presenting with PVT, *P* values were calculated using Fisher exact test.

Majority of our patients had mitral valve replacement (65.93%, n = 60) followed by aortic (17.58%, n = 16) and double valve replacement (mitral + aortic: 16.48%, n = 15); of the 15 cases of double valve replacement, 10 cases of PVT were mitral in location. Hence mitral valve was the location of PVT in 76.9% of the cases (n = 70/91). In 83% of the subjects prosthesis used were bi-leaflet devices followed by tilting disc in 16% and cage-ball in 1%. The average size of the mitral device used was 26.4 ± 1.98 mm and the aortic device was 19.7 ± 2.04 mm. All the patients were on oral anticoagulation therapy, 85 (93.4%) on acenocoumarol and 6 (6.6%) on warfarin. Out of 91 patients, 62 (68%) were females. In 23.3%, the patients reported of previous episodes of PVT and 8.8% of them reported previous history of bleeding gums. Cerebrovascular embolization was noted in 4% of our patients. Both the aortic and mitral prosthetic valve size was smaller in PVT cases, as compared to that of controls (*P* = .005 and *P* = .015 respectively) (Table 1).

They were also investigated for coexisting hypercoagulable state like hypereosinophilia and low cardiac output. A detailed history ruled out pregnancy, autoimmune disease, atrial fibrillation and malignancy, which are associated with prothrombotic status.

Majority of the patients had normal left ventricular function. 47.25% (n = 43) of our study patients were co- prescribed low dose aspirin.

The antiphospholipid antibody screening for Ig M and Ig G antibodies yielded only one positive test for Ig M and none for Ig G and hence not contributory.

### 3.2 Prevalence of allele and genotype frequency distributions of *VKORC1* (-1639G > A), *CYP2C9* (∗1, ∗2 & ∗3) and *CYP4F2* (1347 G > A) polymorphisms

The power of the test is 48% as the size of our study cohort was small and hence is one of the limitations of our study. The result of this study needs to be confirmed on a larger cohort (Table 2).

**Table 2:**
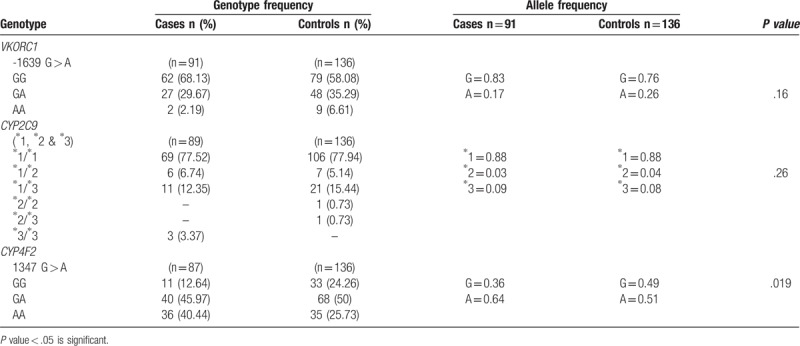
Genotype and allele frequency distributions of *VKORC1* (-1639G > A), *CYP2C9* (^∗^2 & ^∗^3) and *CYP4F2* (1347 G > A) polymorphisms determined using standard frequency analysis and allele counting method, and chi – square test respectively.

The effect size calculation for the genes are; *VKORC1* – 0.45, *CYP2C9* – 0.12 and *CYP4F2* – 0.91.

The genotype distribution for the gene *VKORC1* in the cases showed 68.13% of homozygous wild type (GG) followed by GA (29.67%, n = 27) and AA (2.19%, n = 2). In controls, the allele frequency for G was 0.83 and A was 0.17. The controls had a therapeutic INR and the prevalence for GG was 58.08% followed by 35.29% for GA and 6.61% for AA genotypes (*P* = .16). Though the *P* value was not significant, the OR was calculated as the distribution was similar for the genotypes and also to know the odds of developing PVT when combinations of all the three genotypes were considered.

The control and cases had a similar genotypic distribution for the gene *CYP2C9*. The allele frequency for homozygous wild type (∗1∗1) in cases and controls was found to be 77.52% and 77.94% respectively. Heterozygous genotype ∗1∗2 was 6.74% in cases and 5.14% in controls. ∗1∗3 genotype was found to be 12.35% in cases and 15.44% in controls. The genotype ∗2∗2 (0.73, n = 1) and ∗2∗3 (0.73, n = 1) was found in controls but not in the cases. Though none of the controls had ∗3∗3 genotype, the cases prevailed 3.37% (n = 3) of ∗3∗3 genotype (*P* = .26).

Gene *CYP4F2* had a higher genotype frequency of homozygous wild type (GG) in controls (24.26%, n = 33) to that of the cases (12.64%, n = 11). Whereas homozygous mutant (AA) had a higher genotype frequency in cases (40.44%, n = 36) when compared to the controls (25.73%, n = 35). The allele frequency for G was found to be 0.36 and 0.64 for A in controls (*P* = .019). The patients bearing the homozygous recessive genotype (AA) of *CYP4F2* have an increased incidence of PVT. On calculating the OR, patients bearing A allele showed a 5 fold increased risk of developing PVT (OR = 5.022 (1.39–18.04), *P* = .013) (Table 3, model 3).

**Table 3:**
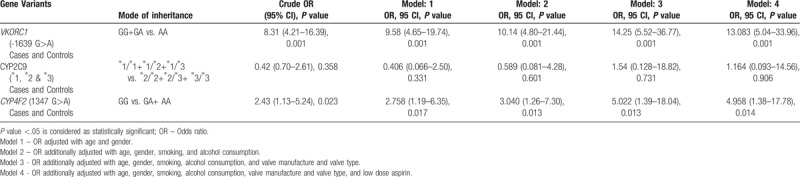
Odds ratio as estimates of relative risk for prosthetic valve thrombosis was calculated using binary logistic regression.

### 3.3 Linkage disequilibrium

Genotype distribution for *VKORC1* C > T 1173 and G > A -1639 were found to be in complete linkage disequilibrium (*r*^2^ = 1. 0, D’ = 1.0, LOD = 102.29) in our study.

### 3.4 International normalized ratio (INR)

At the time of admission, (50.54%, n = 46) of subjects had a sub therapeutic INR (< 2). However, 37.36% (n = 34) of them had a therapeutic INR (2 - 3.5) and 10.98% (n = 10) of the patients were found to have supra therapeutic INR (> 3.5).

## 4 Discussion

A large number of Rheumatic Heart Disease (RHD) patients are treated in our center with valve replacement surgery. Majority of them are young and receive mechanical valves as replacement, owing to their durability and lower reoperation rates, and hence improved patient survival. However, these patients with mechanical valve require lifelong oral anticoagulation therapy to prevent thromboembolic complications. Chronic anticoagulation has the risk of over anticoagulation and bleeding which necessitates close monitoring of the Prothrombin Time (PT) & International Normalized Ratio (INR). Apart from the clinical factors, diet and drug interactions, genetic factors are also known to influence the drug dose - anticoagulant effect response. Among the genetic factors, *VKORC1* and *CYP2C9* polymorphisms are known to be major determinants. Coumarin group of anticoagulants, have a narrow therapeutic range with a wide inter-individual variability and even small dose variations may result in thrombotic or bleeding complications. In spite of the improved hemodynamic design of the mechanical valves and effective oral anticoagulation therapy, patients are still at risk of developing the serious and potentially fatal complication of PVT.

To the best of our knowledge, there has not been any study on the direct association of PVT with *VKORC1* (-1639G > A), *CYP2C9* (∗1, ∗2 & ∗3) and *CYP4F2* (1347 G > A) gene polymorphisms, besides their influence on the anticoagulant. Hence, our study aimed at investigating the role of these genetic factors in the development of PVT.

Approximately 10% of the patients with mechanical valve experience one episode of PVT per year.^[4]^ 23.3% (Table 1) of our patients have reported previous episodes of PVT, of which majority were females (16.48%, n = 15). A literature survey of studies on PVT conducted by Huang et al has shown 59% to 66% of cases occurring in women and our study correlates with 68% (Table 1) incidence in women.^[12]^ However, the reason for female predilection for PVT is not known. INR was found to be sub therapeutic in 50.54% of our patients, the remaining patients had therapeutic and supra therapeutic values. A meta-analysis of previous studies has found 39% of the patients with sub therapeutic INR. However, INR value at the time of admission may not be representative of anticoagulation status at the time of beginning of thrombus formation, as the diagnosis of PVT may be made weeks or months later.^[12]^

We have also observed that patients with smaller prosthetic valves were more prone to develop PVT. The turbulence created by smaller valves is probably responsible for this predilection.

The most prevalent genotype of *VKORC1* -1639 G > A (rs9923231) in our patients was found to be homozygous wild type (GG -68.13%; n = 62) (Table 2). Though there was no statistical significance when compared to the controls, the combination of genotypes when analyzed in various models, carriers of G allele showed a fourteen fold increased risk of developing PVT (OR = 14.25 (5.52 – 36.77), *P* *=* .001) (Table 3, model 3). Our previous study has shown that the carriers of G allele require a higher mean daily dosage of coumarin group of oral anticoagulants to achieve the therapeutic INR. Patients with GG genotype (64.5%) required 2.70 ± 1.04 mg and GA genotype required 2.31 ± 0.90 mg of mean daily acenocoumarol dose.^[10]^

In our study, 75.8% (n = 69) (Table 2) of our patients had wild type (∗1∗1) genotype for *CYP2C9*. However, no significant association was found between *CYP2C9* polymorphism and the risk of development of PVT. Though, the carriers of wild type genotype (∗1∗1) require a higher mean daily dose of coumarin group of anticoagulants to maintain the therapeutic INR.^[13]^

The predominant genotype of *CYP4F2* in our study cohort was heterozygous (43.95%, n = 40) followed by homozygous variants (39.56%, n = 36) (Table 2). The homozygous recessive genotype (AA) of *CYP4F2* was significantly more in our cases (40.44%) compared to the controls (25.73%) (Table 2). The patients bearing allele A exhibited a fivefold increased risk in developing PVT (OR = 5.022 (1.39–18.04), *P* = .013) (Table 3, model 3). On the other hand, in our previous study (unpublished data) we have not found any association with the mean dose requirement of acenocoumarol, while a few studies have found a small but significant association of variant (AA) genotype of *CYP4F2* and increased dose requirement.^[14–16]^

The risk of developing PVT was reduced significantly when the patients were co-prescribed with low dose aspirin (Table 3, model 4) and our findings correlated with recommendation of the guidelines for anticoagulation therapy.^[17]^

Aykan et al^[18]^ found that anticardiolipin (aCL) antibody IgM and Ig G positivity in 14% (n = 16/114) of their patients with PVT. However, only one patient in our study (n = 1/91, 1%) was found to be positive for aCL Ig M and none for aCL Ig G and hence not contributory.

## 5 Limitations

The sample size of our study cohort was small, and the results of this study needs to be confirmed on a larger cohort. The prothrombin time/INR estimation was done for all cases and controls, yet serum acenocoumarol/warfarin levels were not estimated and hence a correlation of our results with drug levels could not be established. However, further large-scale studies in other ethnic populations are warranted.

## 6 Conclusion

The carriers of A allele of *CYP4F2* 1347 G > A polymorphism, had an increased risk of developing PVT in our study. Also, the female patients and patients with a smaller sized prosthetic valve were more predisposed to develop PVT.

## Acknowledgments

We acknowledge Dr. Mariamma Philip, Associate Professor, Department of Biostatistics, NIMHANS, Bengaluru, India; for helping us with the statistical analysis.

## Author contributions

**Conceptualization:** Kalpana SR, Nagaraja Moorthy.

**Data curation:** Bharath G, Simran Jain.

**Formal analysis:** Bharath G, Simran Jain.

**Funding acquisition:** Kalpana SR.

**Investigation:** Kalpana SR, Nagaraja Moorthy, Satvic M, Rita Christopher.

**Methodology:** Bharath G, Simran Jain, Nagaraja Moorthy.

**Project administration:** Kalpana SR.

**Resources:** Kalpana SR.

**Software:** Bharath G, Simran Jain.

**Supervision:** Kalpana SR, Rita Christopher.

**Validation:** Bharath G, Simran Jain.

**Visualization:** Kalpana SR, Nagaraja Moorthy, Rita Christopher.

**Writing – original draft:** Kalpana SR, Bharath G, Simran Jain, Rita Christopher.

**Writing – review & editing:** Kalpana SR, Bharath G, Simran Jain, Nagaraja Moorthy, Satvic M, Rita Christopher.

Satvic M orcid: 0000-0002-1800-8639.
